# EHMTI-0261. Idiopathic intracranial hypertension presenting as acute onset bilateral visual loss

**DOI:** 10.1186/1129-2377-15-S1-C21

**Published:** 2014-09-18

**Authors:** R Handa, RS Jain, K Nagpal, S Prakash, I Bhana, MS Sisodiya, PK Gupta

**Affiliations:** 1 Neurology, SMS Medical College, Jaipur, India

## Introduction

Idiopathic intracranial hypertension (IIH) is a disorder of elevated cerebrospinal fluid pressure of unknown cause. Visual acuity usually remains normal except when the condition is long standing and severe.

## Aims

This case highlights occurrence of acute onset visual loss as a rare presentation of IIH.

## Case report

A 27-years-old obese female, presented with 15 days history of acute onset holocranial throbbing headache and eight days history of acute onset rapidly progressive visual loss in both eyes. Neurological examination revealed bilateral papilloedema with visual acuity 6/60 in right eye and 1/60 in left eye with no other neurological deficit. Contrast enhanced MRI brain was suggestive of IIH and CT venography was normal. CSF manometry showed 520 mm of CSF pressure with normal protein and cellular response. Thus, a final diagnosis of definite IIH was made. Acetazolamide 250 mg thrice a day was started with which she had significant improvement in headache and visual acuity over next 2 weeks and visual acuity was recorded as 6/18 in both eyes at two weeks follow up.

## Conclusion

IIH should always be considered as a possible diagnosis in a patient with headache and papilloedema with no other signs of focal neurological deficits. Although uncommon, acute onset visual loss in IIH can be presenting feature in some patients.

**Figure 1:**
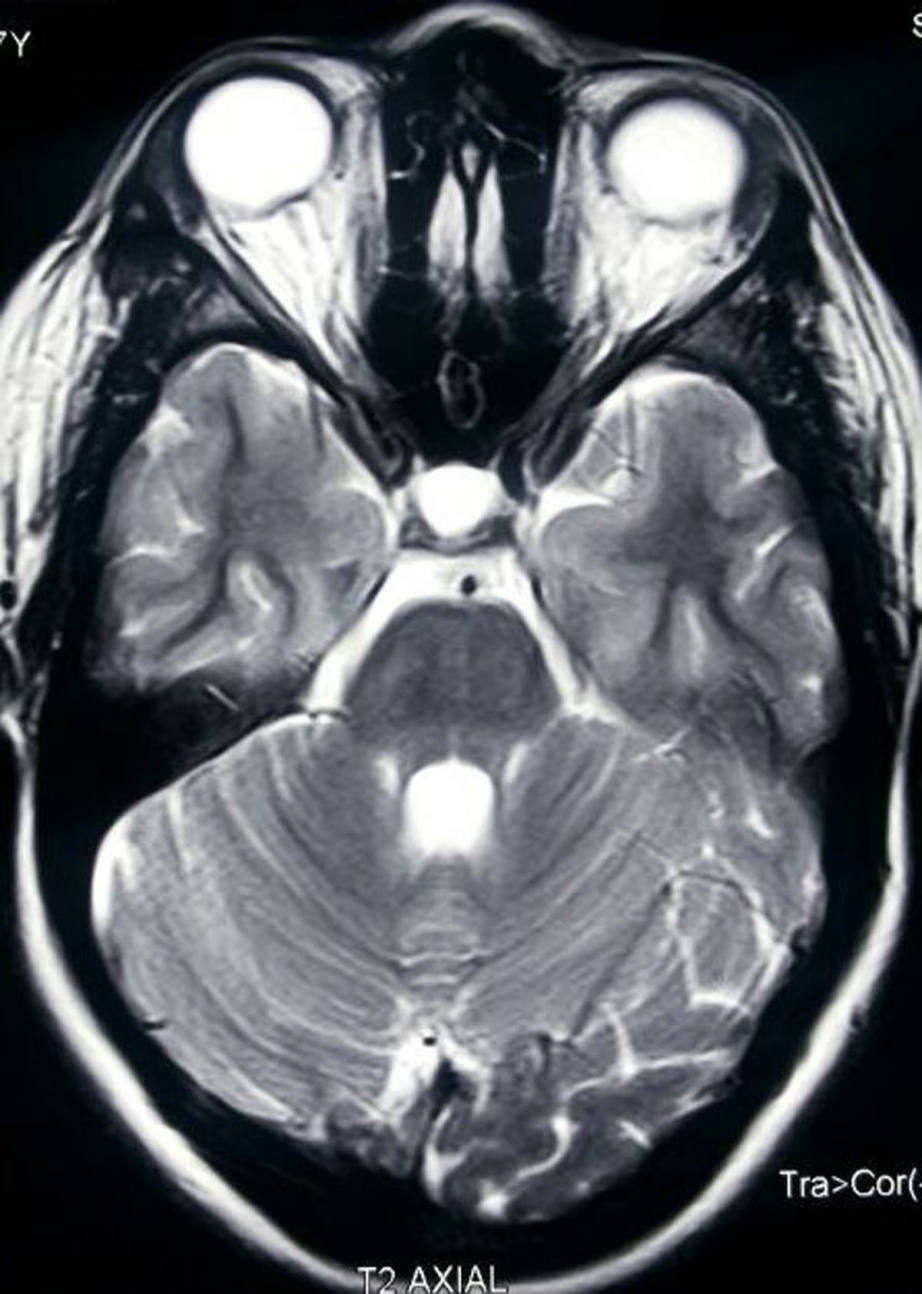
MRI Brain T2W axial image showing empty sella and flattening of posterior aspect of globe.

**Figure 2:**
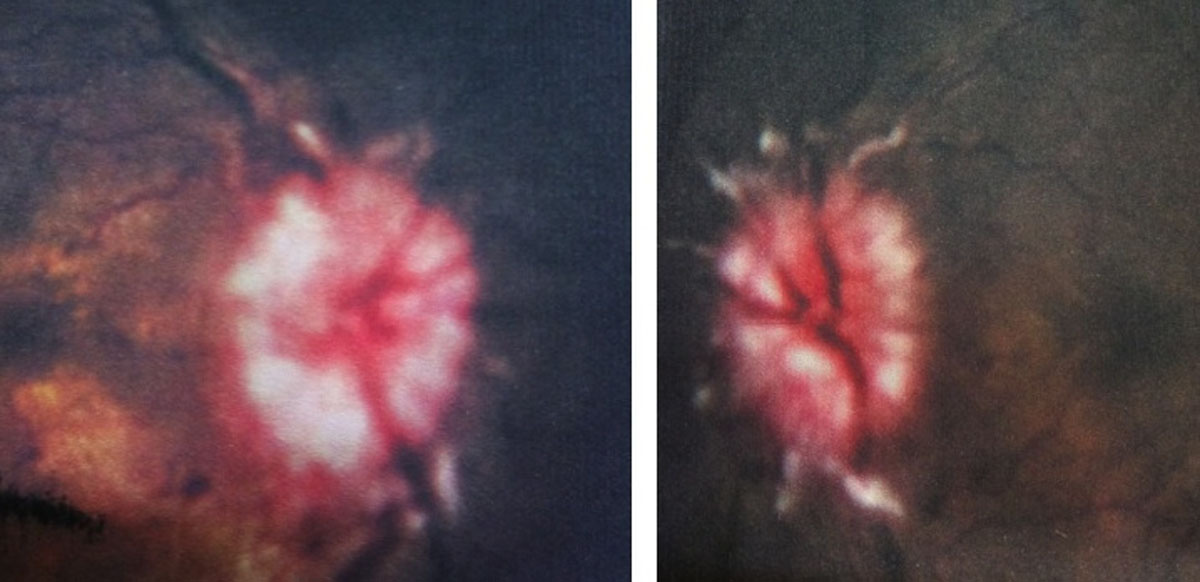
Photograph of fundi showing bilateral papilloedema

No conflict of interest.

